# Co-occurrence of *Methanosarcina mazei* and *Geobacteraceae* in an iron (III)-reducing enrichment culture

**DOI:** 10.3389/fmicb.2015.00941

**Published:** 2015-09-08

**Authors:** Shiling Zheng, Hongxia Zhang, Ying Li, Hua Zhang, Oumei Wang, Jun Zhang, Fanghua Liu

**Affiliations:** ^1^Key Laboratory of Coastal Environmental Processes and Ecological Remediation, Yantai Institute of Coastal Zone Research, Chinese Academy of SciencesYantai, China; ^2^Key Laboratory of Coastal Biology and Biological Resources Utilization, Yantai Institute of Coastal Zone Research, Chinese Academy of SciencesYantai, China; ^3^University of Chinese Academy of SciencesBeijing, China; ^4^Key Laboratory for Genetic Hearing Disorders in Shandong, Binzhou Medical UniversityYantai, China; ^5^The College of Life Sciences, Northwest UniversityXi'an, China

**Keywords:** co-occurrence, *Methanosarcina mazei*, *Geobacteraceae*, direct interspecies electron transfer (DIET), iron(III)-reducing microorganisms

## Abstract

*Methanosaeta harundinacea* and *Methanosarcina barkeri*, known as classic acetoclastic methanogens, are capable of directly accepting electrons from *Geobacter metallireducens* for the reduction of carbon dioxide to methane, having been revealed as direct interspecies electron transfer (DIET) in the laboratory co-cultures. However, whether their co-occurrences are ubiquitous in the iron (III)-reducing environments and the other species of acetoclastic methanogens such as *Methanosarcina mazei* are capable of DIET are still unknown. Instead of initiating the co-cultures with pure cultures, two-step cultivation was employed to selectively enrich iron (III)-reducing microorganisms in a coastal gold mining river, Jiehe River, with rich iron content in the sediments. First, iron (III) reducers including *Geobacteraceae* were successfully enriched by 3-months successive culture on amorphous Fe(III) oxides as electron acceptor and acetate as electron donor. High-throughput Illumina sequencing, terminal restriction fragment length polymorphism (T-RFLP) and clone library analysis based on 16S rRNA genes revealed that the enrichment cultures actively contained the bacteria belong to *Geobacteraceae* and *Bacilli*, exclusively dominated by the archaea belong to *Methanosarcinaceae*. Second, the enrichment cultures including methanogens and *Geobacteraceae* were transferred with ethanol as alternative electron donor. Remarkably, aggregates were successively formed in the enrichments after three transfers. The results revealed by RNA-based analysis demonstrate that the co-occurrence of *Methanosarcina mazei* and *Geobacteraceae* in an iron (III)-reducing enrichment culture. Furthermore, the aggregates, as close physical contact, formed in the enrichment culture, indicate that DIET could be a possible option for interspecies electron transfer in the aggregates.

## Introduction

Methane emission from rivers contributes 1.78–2.26 Tg CH_4_ yr^−1^ to the global budget (0.65 Pg C yr^−1^) of atmospheric CH_4_ which is the second-most important anthropogenic greenhouse gas (Bastviken et al., 2011; Sawakuchi et al., 2014). Microbial activity, in particular that of methanogens is the primary source of methane emission in the river estuary sediments. At moderate temperatures methane is always produced by a combination of acetoclastic methanogenesis involving *Methanosarcinaceae, Methanosaetaceae*, and hydrogenotrophic methanogenesis involving *Methanomicrobiales, Methanobacteriales*, and *Methanocellales* (Rice Cluster I) (Liu and Conrad, 2010). Furthermore, cooperative interactions among microbes belonging to diverse trophic groups are essential for methanogenesis of organic matter (Liu et al., 2009; Liu and Conrad, 2010). In particular, the close syntrophic interaction that is established between hydrogenotrophic methanogens and syntrophic bacteria is regarded as the bottle-neck step of methanogenesis (Liu et al., 2009; Wei et al., 2015).

In recent years, researchers have provided evidence that *Methanosaeta* (Rotaru et al., 2014b) and *Methanosarcina* (Rotaru et al., 2014a) can accept electrons via direct interspecies electron transfer (DIET), suggesting that syntrophy in anaerobic microbiota proceeds not exclusively via the diffusion of electron carriers (e.g., hydrogen and formate) during the conversion of organic matter to methane. As known acetoclastic methanogens, *Methanosaeta* and/or *Methanosarcina* species are often abundant methanogens in many anaerobic digesters and sediments (De Vrieze et al., 2012; van Haandel et al., 2014). Until recently, *Methanosaeta* was considered to have the sole strategy by converting acetate to methane for conserving energy to support growth (van Haandel et al., 2014). However, in laboratory co-cultures both *Methanosaeta harundinacea* (Rotaru et al., 2014b) and *Methanosarcina barkeri* (Rotaru et al., 2014a) reduced carbon dioxide to methane by directly accepting electrons from *Geobacter metallireducens*.

Direct cell contact has been indicated to be required for syntrophic methanogenesis by *Geobacter* and *Methanosaeta* or *Methanosarcina* species (Morita et al., 2011; Rotaru et al., 2014a,b) for interspecies electron transfer (IET). Microbial aggregates, as close physical contact derived from an anaerobic digester treating simulated brewery waste to methane (Morita et al., 2011), were electrically conductive, with a metallic-like conductivity similar to that of *Geobacter* pili (Malvankar and Lovley, 2014). *Geobacter* and *Methanosaeta* species, as the most abundant bacteria and archaea in the aggregates, respectively, were suggested to play important roles in syntrophic methanogenesis coupled to ethanol oxidation (Morita et al., 2011). Furthermore, *M. barkeri* is also reported to be able to accept electrons from *G. metallireducens* through the forming aggregates (Rotaru et al., 2014a). In addition, when hydrogen-producing *Pelobacter carbinolicus* was grown in co-cultures with *M. barkeri*, not aggregates were formed because hydrogen was used as an electron donor for carbon dioxide reduction. These findings, indicate that the close physical contact, such as aggregates, is necessary for DIET but not for interspecies hydrogen transfer.

Electrically conductive substances, including mineral particles (i.e., magnetite) and carbon materials facilitated the syntrophic cooperation, in particular methanogenesis, via DIET (Liu et al., 2012, 2015; Kato et al., 2012a,b; Aulenta et al., 2013; Chen et al., 2014a,b; Cruz Viggi et al., 2014). Magnetite particles have been reported to enhance the methane-production rate from ethanol (Kato et al., 2012a; Liu et al., 2015), propionate (Cruz Viggi et al., 2014), and butyrate (Li et al., 2015) in the enrichments predominated by *Geobacter* and methanogens species from rice paddy field soil or anaerobic digester. Magnetite, as a common mineral in modern soils and sediments (Cornell and Schwertmann, 2003), can originate from both abiotic (Maher and Taylor, 1988; Hochella et al., 2008) and microbial process (Lovley and Phillips, 1987; Yan et al., 2012). Dissimilatory Fe(III)-reducing microorganisms with a wide phylogenetic diversity convert poorly crystalline Fe(III) oxides to abundant extracellular magnetite while using Fe(III) as an electron acceptor for the oxidation of organic compounds or H_2_ (Lovley et al., 2004).

Although the above observations demonstrate that certain methanogens, including *Methanosaeta* and *Methanosarcina* species, can accept electrons from their syntrophic partners (i.e., *Geobacter*) via direct cell contact and conductive materials, whether their co-occurrences are ubiquitous in the iron (III)-reducing environments are still unknown. The objective of our study was to reveal the dominant species in a methanogenic consortium from iron (III)-reducing enrichments. Moreover, we were interested to discover whether other species of acetoclastic methanogens such as *Methanosarcina mazei* are capable of DIET.

## Material and methods

### Sediment samples

**Figure 1:**
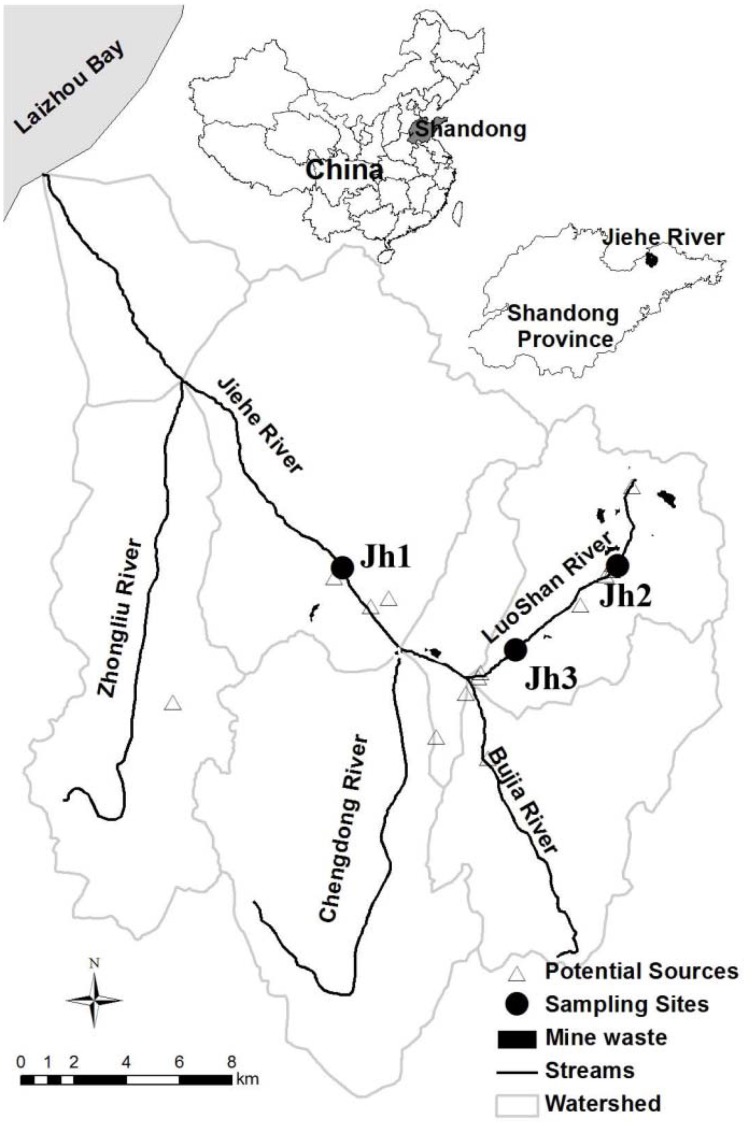
**Location of the sampling points in the Jiehe (Jh) River watershed**. The location of the sediment samples for this study is presented by solid circles (Zhang et al., 2014).

Sediment samples were collected in July 2013 from the Jiehe River (37°05′N-37°33′N, 120°08′E-120°38′E), which is located in the northwest part of the Jiaodong peninsula, China (Figure 1). The geochemical characteristics of stream water were described previously (Zhang et al., 2014). Surface sediment layer (0–5 cm) and the pore water samples were collected using a grab sampler at three sites. Once retrieved, the sediments were homogenized and subsampled within 2 min for DNA analysis. Water samples were subsequently collected with 50-mL sterile centrifuge tubes. After collection, the samples were immediately stored in a refrigerator at 4°C. Within 1 day, the samples were transported cold (4°C) by a car to the Yantai Institute of Coastal Zone Research in Yantai. Characteristics of the sediments for all sampling sites were gravel-like and rich in brownish orange precipitates of iron (III) oxides.

### Chemical analysis

Chemical analyses were conducted in the Public Technology Service Center of Yantai Institute of Coastal Zone Research, Chinese Academy of Sciences. Sediment samples were freeze-dried and passed through a stainless steel sieve (2 mm) before pH, total organic carbon (TOC) and total nitrogen (TN) analysis. pH values were determined for the same sites of whole dried sediments with pH meter by distilled water dilution 1:2.5 (W/V). TOC, TN, and SO^2−^_4_ were determined using elemental analyzer (Vario MACRO cube, Elmental, Germany) and ion chromatography (Dionex ICS-3000, Thermo Fisher Scientific Inc, USA) as the standard procedures. In addition, weak-acid-soluble Fe(II) and total reactive hydroxylamine-reducible iron were extracted from sediments [Fe(II) from enrichment cultures] and each replicate of the assays in triplicate as described previously (Achtnich et al., 1995; Cummings et al., 2000). Briefly, 0.5 g whole sediment (0.5 mL enrichment cultures) was added to 4.5 mL 0.5 mol L^−1^ HCl and incubated overnight at room temperature. Hundred microliter acidified subsample was reacted with ferrozine reagent (0.1% weight ferrozine in 200 mmol L^−1^ HEPES buffer, pH 7.0), and the ferrozine-Fe(II) complex was quantified at 562 nm by UV-Vis spectrophotometer (Thermo Scientific, Genesys 10s UV-Vis, USA). Total extractable Fe was analyzed by reducing the sample to Fe(II) with hydroxylamine hydrochloride (0.25 mol L^−1^), incubated at 60°C for 2 h. After that, the concentration of extractable Fe(III) was determined by the difference between total extractable Fe and Fe(II).

The concentrations of CH_4_ were analyzed using gas chromatography (GC) 7890A (Agilent Technologies, USA) equipped with flame ionization detector (FID). The concentrations of acetate and ethanol were analyzed using high-performance liquid chromatography (HPLC) 1260 Infinity (Agilent Technologies, USA) with a Hi-plexH column equipped with refractive index detector (RID), using 5 mmol L^−1^ H_2_SO_4_ as the eluent. HPLC analyses were performed by separating the organic acids and ethanol.

### DNA extraction and 16S rRNA gene sequencing

DNA of sediment samples was extracted by using a FastDNA™ SPIN Kit for soil (MP Biomedicals, Santa Ana, CA) according to manufacturer's protocol. The quality and concentrations of DNA extracts were determined by standard gel electrophoresis and analyzed by NanoDrop (NanoDrop, Thermo Scientific, USA). PCR was carried out with a Taq DNA polymerase (TaKaRa, Japan) using the universal primer set 519f (CAGCMGCCGCGGTAATWC) and 907r (CCGTCAATTCMTTTRAGTTT) targeting the V4-V5 region of the 16S rRNA. These primers contain Illumina adaptors, and the reverse one was encoded with 5 bp barcodes. Triplicate reaction mixtures per sample were pooled, purified using the QIAquick PCR Purification kit (QIAGEN), and normalized in equimolar amounts before pyrosequencing by means of a MiSeq sequencer (Illumina). Raw reads of the bacterial 16S rRNA gene were processed using Trimmomatic (Bolger et al., 2014) and FLASH (Reyon et al., 2012) to merge the paired-end reads. The low quality sequences were filtered and chimeric sequences were removed by using USEARCH (Edgar et al., 2011). Sequences were clustered into operational taxonomic units (OTUs) using CD-HIT (Li and Godzik, 2006) with a cut-off of 97% sequence identity, and the most abundant sequence from each OTU was selected as a representative sequence for that OTU. The taxonomy of OTU representative sequences at the genus level were phylogenetically assigned to taxonomic classifications by RDP Classifier (Wang et al., 2007) and Greengenes database (Desantis et al., 2006). Sequences related to putative dissimilatory iron-reducing bacteria were selected according to published reviews (Weber et al., 2006; Lovley, 2013).

### Enrichment and isolation

To enrich for iron(III)-reducing microorganisms, a fresh water enrichment (FWE) medium was provided with 100 mmol L^−1^ Fe(III) oxides as electron acceptor and 33 mmol L^−1^ acetate as electron donor (Lovley and Phillips, 1986). Iron (III)-reducing microorganisms were grown in DSMZ methanogenic medium 120 with 30 mmol L^−1^ ethanol as electron donor after three transfers of the acetate-fed enrichment cultures by 10% inoculation. The medium (40 mL) was added to 100 mL serum bottles under an atmosphere of N_2_/CO_2_(80/20, V/V). Bottles were capped with butyl rubber stoppers and aluminum seals. After autoclaving, the sludge sample (4 g of wet weight) was inoculated into 100 mL sealed serum bottles containing 40 mL culture medium and incubated at 30°C in the dark without shaking. For each sample of enrichment culture, the vials were performed in triplicate for RNA extraction and for measurement of CH_4_, ferrous iron, ethanol, acetate.

For isolation, sediment samples after incubation were transferred as 10% inoculums into fresh media. Iron(III)-reducing microorganisms were grown in FC (Bagnara et al., 1985) and NBF medium (Deppenmeier et al., 1988). For FC medium, 20 mmol L^−1^ acetate was added as the sole electron donor and 55 mmol L^−1^ Fe(III) citrate as the electron acceptor. For NBF medium, 10 mmol L^−1^ acetate was added as the sole electron donor and 40 mmol L^−1^ fumarate as the electron acceptor. Methanogens were grown in DSMZ methanogenic medium 120 (Lueders and Friedrich, 2002) with 30 mmol L^−1^ acetate. 10% inoculums of the highest dilution (up to 10^−8^) were transferred into the liquid medium with 2.0% agar and used by the Hungate roll-tube technique (Bagnara et al., 1985). After 2 weeks, colonies were selected individually in an anaerobic chamber and transferred into liquid medium.

### Nucleic acids extraction and gene amplification

RNA extraction of enrichment cultures was performed using a bead-beating protocol with modifications as previously described (Shrestha et al., 2009). Briefly, once enrichment cultures (30 days) were harvested, iron particles in the pellet were dissolved by addition of filter-sterilized oxalate solution (197 mmol L^−1^ ammonium oxalate and 119 mmol L^−1^ oxalic acid) to separate cells from the iron floc. After that, cells were extracted firstly with cool TPM buffer (50 mmol L^−1^ Tris-HCl pH 7.0, 1.7% polyvinylpyrrolidone, 20 mmol L^−1^ MgCl_2_) and then with PBL buffer (5 mmol L^−1^ Tris-HCl pH 7.0, 5 mmol L^−1^ Na_2_EDTA pH 8.0, 0.1% SDS, 6% water-saturated phenol). Bead-beating was performed in BeadBeater-16 (Biospec, USA). The supernatants were extracted with water-saturated phenol, phenol-chloroform-isoamyl alcohol (25:24:1), and chloroform-isoamyl alcohol (24:1), respectively. The extracts of nucleic acids were precipitated, washed and stored at −80°C or processed immediately. Total nucleic acids were treated with gDNA Eraser (TaKaRa, Japan) to remove co-extracted DNA. RNA was confirmed to be DNA-free by the absence of PCR products by amplifying 16S rRNA genes with universal primers Ba27f (5′-AGA GTT TGA TCC TGG CTC AG-3′) and Ba907r (5′-CCG TCA ATT CCT TTR AGT TT-3′) for the bacteria, Ar109f (5′-ACK GCT CAG TAA CAC GT-3′) and Ar915r (5′-GTG CTC CCC CGC CAA TTC CTT TA-3′) for the archaea.

To synthesize cDNA, reverse transcription was performed according to PrimeScript™ RT reagent Kit (TaKaRa, Japan) after RNA denaturation at 70°C for 10 min, followed by a incubation step at 37°C for 50 min.

For PCR amplification, bacterial and archaeal 16S rRNA gene were amplified from community cDNA or DNA of sediments with the primers Ba27f/Ba907r and Ar109f/Ar915r. Amplification was performed with a Mastercycler pro S (Eppendorf) starting with 2 min at 94°C, followed by 30 cycles consisting of denaturation 30 s at 94°C, annealing 30 s at 55°C, extension 1 min at 72°C, and a final extension at 72°C for 10 min.

### T-RFLP analysis

T-RFLP was performed as described previously (Liu and Conrad, 2011). T-RFLP analysis of rDNA and rRNA were from triplicate DNA and RNA extractions for each sample. Bacterial/archaeal communities T-RFLP analysis were performed using primers Ba27f/Ba907r and Ar109f/Ar915r with Ba27f and Ar915r were labeled at the 5′ end and 3′ end with 6-carboxyfluorescein (FAM), respectively. Briefly, to minimize the PCR artifacts, the PCR amplification procedure was modified: 20 ng of template DNA, doubled concentration of primer and 25 amplification cycles of PCR were used as described previously in detail (Zhang et al., 2005). Fluorescently labeled PCR products were purified and digested using *Msp*I(Fermentas) for bacteria and *Taq*I(Fermentas) for archaea, respectively, and subsequently analyzed using an automated sequencer ABI PRISM 3730XL (Applied Biosystems). T-RFLP patterns of each sample were evaluated by peak height integration of the different terminal restriction fragments (T-RFs) using GeneMapper 4.1 analysis software (Applied Biosystems). The relative abundance of T-RFs was calculated as described previously (Ma et al., 2012) as the percentage of one distinct T-RF in the sum of all peak heights in an individual T-RFLP profile. Statistical analyses were performed using SigmaPlot for Windows Version 11.0 (systat software, Inc., USA).

### Sequencing and phylogenetic analysis

Clone libraries of 16S rRNA genes were constructed from community DNA of sediments and cDNA of acetate or ethanol grown cultures. Amplicons were cloned into *Escherichia coli* DH5α using pMD18T vector system (TaKaRa) according to the manufacturer's instructions. From each library, randomly selected clones were screened for positive inserts by PCR with the M13 primers and sequenced at Life Technologies (Beijing, China). Nucleotide sequences were analyzed with DNAStar 7.0 (Madison, WI, USA), almost full length sequences were aligned online using SINA Aligner (http://arb-silva.de/aligner) from the SILVA bacterial and archaeal 16S rRNA gene database project. Sequences of the clone library were analyzed with MOTHUR v1.32 (http://www.mothur.org/) (Schloss et al., 2009) by defining operational taxonomic units (OTU), in which representative sequences from each OTU was defined by 97% sequence identity. Close relatives and taxonomic assignments were checked using BLAST searches on the Greengenes website (http://greengenes.lbl.gov/cgi-bin/nph-index.cgi). All sequences were digested *in silico* using DNAMAN 8.0 (LynnonBiosoft, USA). Phylogenetic analysis of the sequences from bacterial and archaeal clone libraries was performed using the MEGA 6.0 (Tamura et al., 2013) software package with neighbor-joining method. Sequence data have been submitted to the GenBank database under accession numbers KT008225 to KT008243 for bacteria and KT008244 to KT008264 for archaea, respectively.

## Results

### Diversity of microorganisms in sediments

**Figure 2:**
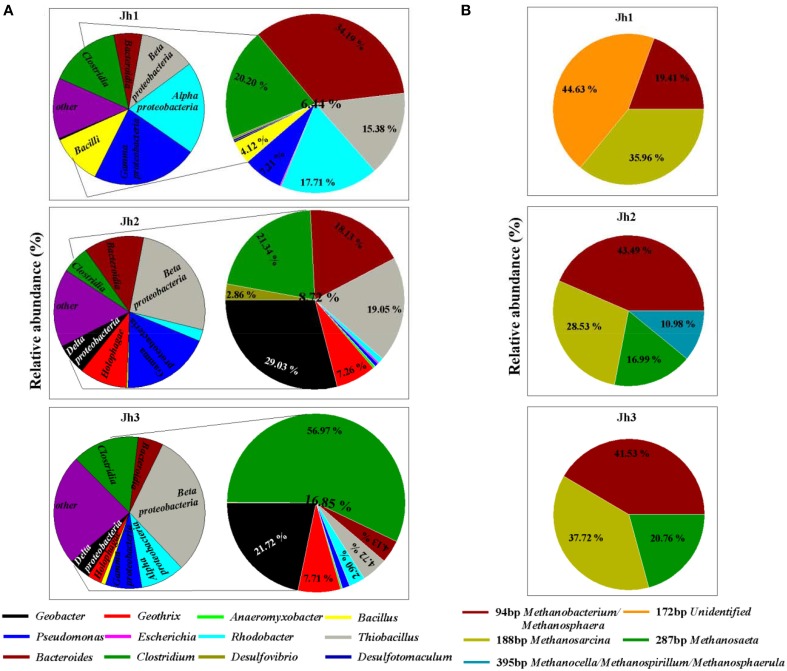
**Relative abundance of bacteria and archaea in *in situ* three sediment samples**. **(A)** Relative abundance of iron(III)-reducing bacteria as determined by Illumina sequencing according to published reviews. The left side of the figure chart represents iron(III)-reducing related bacteria at the class level, whereas its right side of the larger figure chart gives an estimate of iron(III)-reducing related bacteria as represented at the genus level. **(B)** Relative abundance of archaea in three sediment samples as determined by T-RFLP.

High-throughput Illumina sequencing was used to reveal the diversity of iron (III)-reducing microorganisms and methanogens in the iron-rich sediments of a coastal gold mining river, Jiehe River. The characteristics of the sediment samples from Jiehe River were summarized in Table 1 and the microbial diversity were shown in Figure 2, Figure S2. A total of 92,535 bacterial sequences (mean = 10,281 sequences per library; median = 10,081; maximum = 12,934; minimum = 6806) were analyzed, and the number of OTUs varied between 593 and 1426 with a 97% identity threshold. The rarefaction curves of three samples were shown in Figure S1. The overall phylum-level phylogenetic characteristics from the heatmap diagram (Figure S2A) and the 100% stacked column chart (Figure S2B) indicated that *Proteobacteria, Firmicutes, Bacteroidetes*, and *Acidobacteria* were the dominant bacteria in these sediment samples. In the *Proteobacteria, Deltaprotebacteria* dominated the three libraries, accounting for 0.36, 6.67, and 4.69% of the community in Jh1, Jh2, and Jh3, respectively. Within the *Deltaprotebacteria, Geobacteraceae* represented the highest relative abundance accounting for 2.57 and 3.67% of the community in Jh2 and Jh3 respectively. *Geobacter* spp., *Anaeromyxobacter* spp., *Desulfovibrio* spp. of *Deltaproteobacteria*, and *Geothrix* spp. of *Acidobacteria* (Figure 2A) are all known dissimilatory iron(III)-reducing microorganisms. In the *Firmicutes, Bacteroidetes* and *Proteobacteria*, the majority of the sequences belong to the genus *Clostridium* spp., *Bacillus* spp., *Desulfotomaculum* spp., *Bacteroides*, and *Pseudomonas* spp., *Escherichia* spp., *Rhodobacter* spp., and *Thiobacillus* spp. All the sequences affiliated to known dissimilatory iron(III)-reducing bacteria accounted for 6.44, 8.72, and 16.85% of the community in Jh1, Jh2, and Jh3, respectively. Remarkably, *Geobacter* spp. and *Clostridium* spp. dominated in the dissimilatory iron(III)-reducing bacteria of Jh2 and Jh3 accounting for 29.03 and 56.97%, respectively (Figure 2A). Furthermore, the results of T-RFLP showed that iron(III)-reducing bacteria in three sediment samples were accompanied by the archaea belonging to *Methanosarcina, Methanobacterium/Methanosphaera, Methanosaeta*, and *Methanocella/Methanospirillum/Methanosphaerula* (Figure 2B). Jh2 was selected as the representative sample for further experiments named as Jh.

**Table 1:** **Physico-chemical characteristics of the sediment samples**.

**Site**	**Color and composition**	**pH**	**EC (mS cm^−1^)^a^**	**Total organic carbon**	**Total nitrogen**	**Fe(II)**	**Fe(III)^d^**	**Total iron**	**Sulfate (SO^2−^_4_)**
				**[% (DW^e^)]^b^**		**[mg g (DW^e^)^−1^]^c^**			**[mg g (DW^e^)^−1^]^f^**
Jh1	Gray, fine grained sand	8.71	2.69	0.529	0.125	0.73(0.01)	5.71(0.47)	6.44(0.48)	1.52(0.076)
Jh2	Brownish orange, larger sand	5.84	4.70	0.525	0.112	1.08(0.04)	13.81(0.05)	14.89(0.09)	1.02(0.051)
Jh3	Black, silt	6.36	1.61	0.848	0.171	1.33(0.11)	5.53(0.21)	6.86(0.32)	0.87(0.044)

a *Electrical conductivity (EC), analyzed in duplicates*.

b *Measurement by Elemental Analyzer*.

c *Average of triplicates (±standard deviation) by the ferrozine assay*.

d *Calculated from total iron and Fe(II)*.

e *Dry weight sediment*.

f *Measurement by ion chromatography*.

### Iron(III) reduction and methane production with aggregation

**Figure 3:**
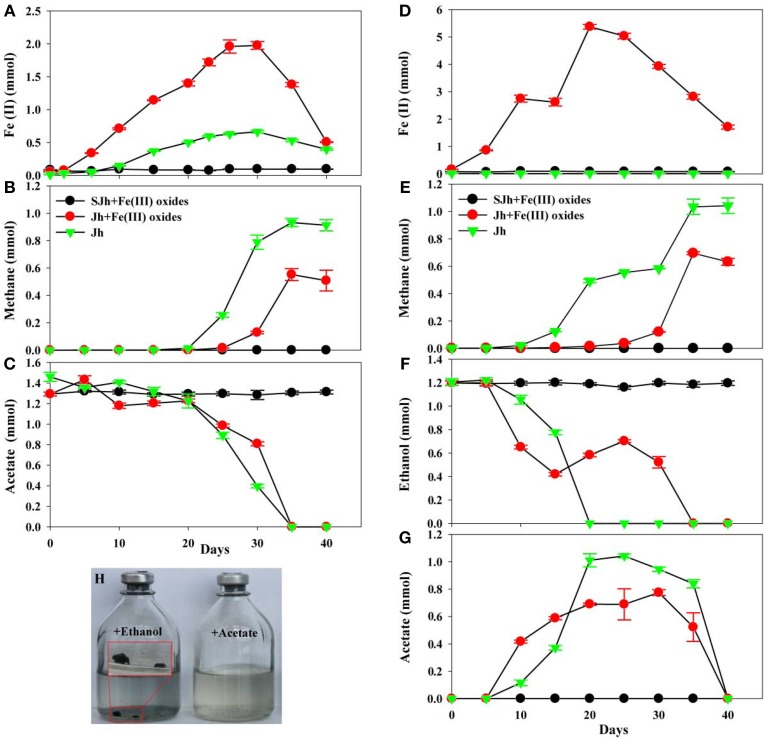
**Fe(III) reduction, methane production and acetate or ethanol consumption of enrichment cultures**. Time-courses of Fe(II) production **(A)**, methane production **(B)** and acetate consumption **(C)** in enrichment cultures by 10% (w/v, wet) inoculation from fresh water enrichment culture of *in situ* sediments with 33 mmol L^−1^ of acetate in the absence or presence of 100 mmol L^−1^ of Fe(III) oxides. Time-courses of Fe(II) production **(D)**, methane production **(E)**, ethanol consumption **(F)** and acetate production/consumption **(G)** in enrichment cultures by 10% (v/v) inoculation from enrichment culture of three transfers of *in situ* sediments in DSM 120 medium with 30 mmol L^−1^ of ethanol in the absence or presence of 100 mmol L^−1^ of Fe(III) oxides. **(H)** Image was presented aggregates after enrichment culture in DSM 120 medium with ethanol or acetate as a substrate in the absence of Fe(III) oxides. Sterile sediment sample (SJh) with Fe(III) oxides treatment was used as the negative control. Data was presented in triplicate and standard deviation was shown for each data point. Jh2 was selected as the representative sample named as Jh.

In order to investigate whether DIET is involved between more iron(III)-reducing microorganisms and methanogens, syntrophic co-cultures systems need to be set up. Instead of initiating the co-cultures with pure cultures, two-step cultivation was employed to selectively enrich iron (III)-reducing microorganisms and methanogens from the sediments. First, iron (III) reducers were enriched by 3-months successive culture on amorphous Fe(III) oxides as electron acceptor and acetate as electron donor. Second, the enrichment cultures were transferred with ethanol as alternative electron donor. During the first step, ferrous iron was produced during the enrichment with or without Fe(III) oxides treatment. Under the Fe(III) oxides-amended conditions with acetate as electron donor, 1.98 ± 0.059 mmol of ferrous iron was produced at day 30 (Figure 3A). When the enrichment culture was transferred for three times, ethanol was used as electron donor. As a result, 5.37 ± 0.086 mmol of ferrous iron was produced at day 20 (Figure 3D). However, when acetate was used as the electron donor without Fe(III) oxides treatment, only 0.66 ± 0.018 mmol of ferrous iron was produced at day 30 (Figure 3A), while the amount of ferrous iron was below 0.012 mmol in the culture with ethanol as the electron donor after three transfers (Figure 3D). Thus, part of the ferric iron in acetate or ethanol-fed cultures, mostly consisting of the supplemented Fe(III) oxides, was reduced to ferrous iron. As a control to show this reduction is a biogeochemical process, autoclaved sediments did not produce any more ferrous iron (Figures 3A,D).

Under the acetate or ethanol-amended conditions, methane production was largely suppressed in the presence of supplemented Fe(III) oxides during the incubation period compared with that without Fe(III) oxides. The amount of methane in the Fe(III) oxides treatment increased only slightly to 0.016 ± 0.0005 mmol after 25 days whereas rapidly to 0.55 ± 0.043 mmol after 35 days in acetate-fed cultures (Figure 3B). While in cultures without treatment, the amount of methane increased to 0.93 ± 0.03 mmol after 35 days (Figure 3B), which is higher than that of treatment with Fe(III) oxides. Similarly, under the ethanol-amended conditions, the amount of methane in the Fe(III) oxides treatment increased to 0.70 ± 0.01 mmol after 35 days. While without treatment, the amount of methane increased to 1.04 ± 0.057 mmol after 40 days (Figure 3E) incubation.

The decline of acetate was more rapid in the presence of Fe(III) oxides than that without Fe(III) oxides at the beginning of 20 days, however, the rate slow down afterwards, and correspondingly ferrous iron increased and then following methane increased (Figure 3C). Calculations suggest that in Fe(III) oxides cultures, 18.8 and 41.7% of the electrons from acetate oxidation were recovered in ferrous iron and methane, respectively. While in the culture without Fe(III) oxides, 6.3, 70.5% of the electrons from acetate oxidation were recovered in ferrous iron and methane, respectively. Similarly, acetate only transiently accumulated in enrichment with the decline of ethanol as the electron donor (Figures 3F,G), and then subsequently decreased with methane production. The calculation suggests that in the cultures with Fe(III) oxides amendment, 58.3% of the electrons from ethanol oxidation were recovered in methane, while in the cultures without Fe(III) oxides cultures, 86.7% of the electrons from ethanol oxidation were recovered in methane. Remarkably, aggregates were found in the transferred cultures with ethanol as electron donor (Figure 3H).

### Bacterial community

**Figure 4:**
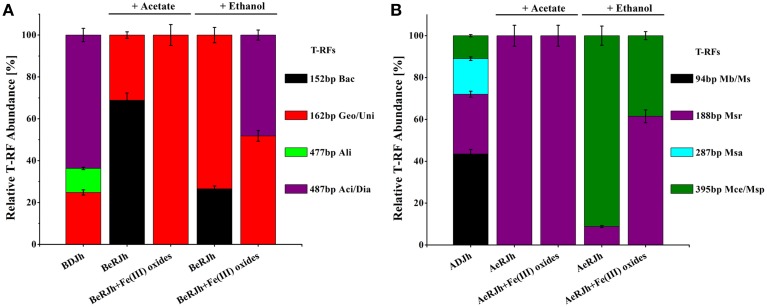
**Community characteristics of bacteria and archaea in the sediments before and after the enrichment incubations, as revealed by T-RFLP and clone libraries of 16S rRNA genes**. **(A)** Relative abundance of different bacterial T-RFs from DNA and RNA extracted from *in situ* sediments and enrichment cultures (with or without Fe(III) oxides treatment). T-RF fingerprints were generated using *Msp* I restriction enzyme. Bac-*Bacillus*; Geo-*Geobater*; Uni-*Unidentified*; Ali-*Alishewanella*; Aci/Dia-*Acidovorax/Diaphorobacter*. **(B)** Relative abundance of different archaeal T-RFs from DNA and RNA extracted from *in situ* sediments and enrichment cultures. T-RF fingerprints were generated using *Taq* I restriction enzyme. Mb/Ms-*Methanobacterium/Methanosphaera*; Msr-*Methanosarcina*; Msa-*Methanosaeta*; Mce/Msp-*Methanocella/Methanospirillum/Methanosphaerula*. T-RF size in base pairs. BDJh, Bacterial DNA extracted from sediment Jh; BeRJh, Bacterial RNA extracted from enriched sediment Jh; ADJh, Archaeal DNA extracted from sediment Jh; AeRJh, Archaeal RNA extracted from enriched sediment Jh. Relative T-RF abundance of rDNA and rRNA were presented from triplicate DNA and RNA extractions for each sample.

Analysis of the relative abundance of the bacterial terminal restriction fragments (T-RF) after an incubation period is shown in Figure 4A. For rRNA (enrichment cultures of 30 days) with Fe(III) oxides treatment and acetate as the electron donor, the T-RF of 162 bp was exclusively predominant in enrichment cultures. After 3-months successive culture, rRNA with Fe(III) oxides treatment and ethanol as the electron donor, the T-RF of 162 and 487 bp were predominant in enrichment cultures. For rRNA without Fe(III) oxides treatment (enrichment cultures of 30 days), the T-RF of 152 and 162 bp were predominant in enrichment cultures with acetate or ethanol as the electron donor. For rDNA without Fe(III) oxides treatment, the T-RF of 162, 477, and 487 bp were predominant in sediments.

**Figure 5:**
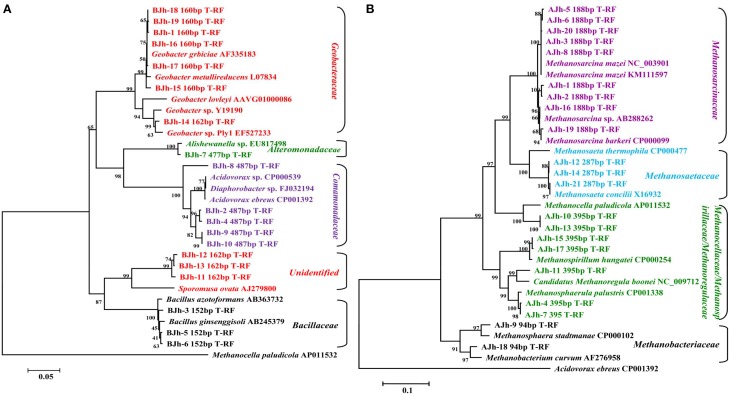
**Neighbor-joining phylogenetic tree of representative bacterial (A) and archaeal (B) 16S rRNA gene clones generated from DNA and RNA extracted from *in situ* sediments and enrichment cultures**. Numbers of T-RF lengths are shown in base pairs. An association with the sequence of the highest similarity in the database and GenBank accession number of reference sequence as indicated. The scale bars represents 5% **(A)** and 10% **(B)** sequence divergence, respectively. *Methanocella paludicola* and *Acidovorax ebreus* were selected as outgroups of bacterial **(A)** and archaeal **(B)** phylogenetic tree, respectively.

In order to affiliate the detected T-RFs to phylogenetic bacterial groups, four clone libraries were generated using the samples. The phylogenetic affiliations of all bacterial clones analyzed were summarized in Table 2. The phylogenetic placement of selected representative clones was shown in Figure 5A.

**Table 2:** **Phylogenetic affiliation of 16S rRNA sequences retrieved in clone libraries generated from three different sites with major bacterial lineages of clones falling into major MspI-specific T-RF classes**.

**OTU^a^**	**Phylogenetic group^b^**	**BDJh**		**BeRJh^d^**		**BeRJh** + **Fe(III) oxides^d^**		**BeRJh^e^**	
		**No**.	**T-RF (bp)^c^**	**No**.	**T-RF (bp)^c^**	**No**.	**T-RF (bp)^c^**	**No**.	**T-RF (bp)^c^**
	***Firmicutes***								
	*** Bacillaceae***								
BJh-3	* Bacillus*	1	145	2	152	4	150,152		
	***Proteobacteria***								
	***Betaproteobacteria***								
	*** Comamonadaceae***								
BJh-2	* Diaphorobacter/Acidovorax*	12	485,486,487	4	487,488	1	487		
	***Gammaproteobacteria***								
	*** Alteromonadaceae***								
BJh-7	* Alishewanella*	1	477						
	***Deltaproteobacteria***								
	*** Geobacteraceae***								
BJh^d^-14, BJh^d^-15, BJh^d^-16	* Geobacter*	8	145,160,162	2	162	10	160		
BJh^e^-11	* Unidentified*							13	162
	*** Other Bacteria***	55	90,279,521,527,535	12	90,139,517,522	3	90,155	5	158,295, 490,537
	Total(Clones)133	77		20		18		18	

a *An OTU consist of sequences exhibiting ≥97% sequence identity using Mothur software*.

b *Based on BLAST tool (http://greengenes.lbl.gov)*.

c *Terminal restriction fragment (T-RFs) length were shown in base pairs (bp, MspI) for different clones*.

d *Represented a clone library of enrichment culture with acetate*.

e *Represented a clone library of enrichment culture with ethanol*.

*Bold: at family-level or above*.

We used the sequence data to tentatively assign major T-RFs observed in different bacterial fingerprints to defined phylogenetic lineages. Thus, the predominant 162 bp T-RF represented members of *Geobacteraceae*, which dominated the rRNA clone library from the incubation treated with Fe(III) oxides. In contrast, the predominant 152, 477, and 487 bp T-RFs represented members of *Bacilli, Alteromonadaceae*, and *Comomonadaceae*, respectively.

### Archaeal community

Analysis of the relative abundance of the archaeal terminal restriction fragments (T-RF) after an incubation period is shown in Figure 4B. For rRNA (enrichment cultures of 30 days) with or without Fe(III) oxides treatment and acetate as electron donor, the T-RF of 188 bp was exclusively predominant in enrichment cultures. After 3-months successive culture, rRNA (enrichment cultures of 30 days) with or without Fe(III) oxides treatment and ethanol as the electron donor, the T-RF of 188 and 395 bp were predominant in enrichment cultures. For rDNA without Fe(III) oxides treatment, the T-RF of 94, 188, 287, and 395 bp were predominant in sediments.

In order to affiliate the detected T-RFs to phylogenetic archaeal groups, four clone libraries were generated using the same samples. The phylogenetic affiliations of all bacterial clones analyzed were summarized in Table 3. The phylogenetic placement of selected representative clones was shown in Figure 5B.

**Table 3:** **Phylogenetic affiliation of 16S rRNA sequences retrieved in clone libraries generated from three different sites with major archaeal lineages of clones falling into major TaqI-specific T-RF classes**.

**OTU^a^**	**Phylogenetic group^b^**	**ADJh**		**AeRJh^d^**		**AeRJh** + **Fe(III) oxides^d^**		**AeRJh^e^**	
		**No**.	**T-RF (bp)^c^**	**No**.	**T-RF (bp)^c^**	**No**.	**T-RF (bp)^c^**	**No**.	**T-RF (bp)^c^**
	***Euryarchaeota***								
	***Methanomicrobia***								
	*** Methanosarcinaceae***								
AJh-8, AJh-19	* Methanosarcina*	18	188	10	188	9	188	11	188
	*** Methanosaetaceae***								
AJh-21	* Methanosaeta*	7	287						
	***Methanocellaceae/Methanospirillaceae/***								
	***Methanoregulaceae***								
AJh-10,	* Methanocella/*								
AJh-17,	* Methanospirillum/*	7	395					1	395
AJh-4	* Methanosphaerula*								
	***Methanobacteria***								
	*** Methanobacteriaceae***								
AJh-18,	* Methanobacterium/*	12	94						
AJh-9	* Methanosphaera*								
	***Other Archaea***	7	226,681,723,741					3	383,680
	Total(Clones)85	51		10		9		15	

a *An OTU consist of sequences exhibiting ≥ 97% sequence identity using Mothur software*.

b *Based on BLAST tool (http://greengenes.lbl.gov)*.

c *Terminal restriction fragment (T-RFs) length were shown in base pairs (bp, TaqI) for different clones*.

d *Represented a clone library of enrichment culture with acetate*.

e *Represented a clone library of enrichment culture with ethanol*.

*Bold: at family-level or above*.

We used the sequence data to tentatively assign major T-RFs observed in different archaeal fingerprints to defined phylogenetic lineages. Thus, the predominant 188 bp T-RF represented members of *Methanosarcinaceae*, which dominated the rRNA clone library from the incubation treated with or without Fe(III) oxides. In contrast, the predominant 94, 287, and 395 bp T-RFs represented members of *Methanobacteriaceae, Methanosaetaceae*, and *Methanocellaceae/Methanospirillaceae/Methanoregulaceae*, respectively.

### Enrichment and isolation

In order to get the native iron (III)-reducing microorganisms for heavy metals bioremediation in a coastal gold mining river, Jiehe River, FC and NBF medium was used for enrichment and isolation. Six Fe(III)-reducing bactetia were isolated in FC media with acetate as electron donor. Sequencing analysis of 16S rRNA gene indicated that all strains affiliated with the species of *Clostridium* spp. (FN397991) with 97.41% identity, this closest cultivated relative is *Clostridium* sp. AN-D, which was isolated from gas hydrate containing sediments (Parkes et al., 2009). Two methanogenic isolates were isolated in DSMZ methanogenic medium 120 with acetate as electron donor, these two strains exhibited 98.77% similarity to the species of *Methanosarcina mazei* (NC_003901), this closest cultivated relative is mesophilic archaeon *Methanosarcina mazei* strain Gö1 (Deppenmeier et al., 1988, 2002).

## Discussion

*Methanosaeta harundinacea* and *Methanosarcina barkeri* can make direct electrical connections with *Geobacter metallireducens*, accepting electrons for the reduction of carbon dioxide to methane, having been known as DIET (Rotaru et al., 2014a,b). Our results showed that the co-occurrence of another species of *Methanosarcina, Methanosarcina mazei*, and *Geobacteraceae* in an iron(III)-reducing microbial community from a coastal gold mining river, Jiehe River, forming aggregates with ethanol as electron donor, indicate that DIET could be a possible option for interspecies electron transfer in the aggregates.

*M. mazei* Gö1, which belongs to the mesophilic methanogenic archaea, was isolated from sewage sludge in Göttingen, Germany (Deppenmeier et al., 1988). It is able to convert H_2_ plus CO_2_, methanol, acetate or methylamines to methane. Its ability to use a wide spectrum of carbon and nitrogen sources reflects the organism's high ecological relevance. Moreover, *M. mazei* alone is capable of reducing structural Fe(III) in illite-smectite minerals (Zhang et al., 2012). Therefore, a further study on the interactions between *Methanosarcina mazei* and *Geobacteraceae* in a defined co-culture system is expected.

As anaerobically respiring microorganisms, dissimilatory iron reducers are widespread, comprising many bacterial lineages and members of the archaea (Lovley et al., 2004; Weber et al., 2006). However, tracking of them in the environment is rather difficult because no universal functional gene markers are available so far (Hori et al., 2010). *Geobacter* spp. have been intensively studied as a model in varied environments, such as mining-impacted lake sediments (Cummings et al., 2000), heavy metal and radionuclide contaminated river (Scala et al., 2006). *Clostridium* spp. was also often found in the iron-reducing environments (Shcherbakova et al., 2005; Porsch et al., 2009; Suetin et al., 2009), playing an important role in hydrogen production as the substrate for subsequent hydrogenotrophic methanogenesis (Jiang et al., 2013).

Significant numbers of methanogens have also been found in the Fe(III) oxides rich sediments. For instance, in wetlands, river sediments and rice paddy soils, when flooded methanogenic soils are dried and subsequently exposed to air, Fe(II) in the soil is oxidized back to Fe(III) (Bond and Lovley, 2002). Methanogens survive in such sediments or soils (Yao and Conrad, 1999) and become metabolically active again when the anaerobic conditions redevelop. *Methanosarcina, Methanosaeta, Methanobacterium*, and *Methanomicrobium* species dominated in the methanogenic community of sediment cores of River Sitka, Czech Republic (Mach et al., 2015). *Methanomicrobiales* and *Methanosarcinales* predominated in the mud flat sediments of Yangtze River estuary, China (Zeleke et al., 2013). *Methanosarcinales*/*Methanosaetaceae* decrease with depth in relative abundance while *Methanomicrobiales* increase in fresh water systems (Chan et al., 2005; Zeleke et al., 2013).

Although studies indicate that *Methanosaeta* and *Methanosarcina* species can accept electrons from their syntrophic partners such as *Geobacter* via direct cell contact within aggregates and conductive materials such as magnetite, the molecular mechanisms mediating electron uptake by these methanogens are largely unknown (Kouzuma et al., 2015). A deeper understanding of the effect of magnetite formation coupled with iron (III)-reduction on potential DIET will not only help elucidate syntrophic microbial behavior under methanogenic conditions, but will also provide novel strategies for renewable bioenergy processes with high efficiency.

In summary, the co-occurrence of *Methanosarcina mazei* and *Geobacteraceae* and their activities in an iron(III)-reducing microbial community from a coastal gold mining river, Jiehe River, has been found and confirmed by culture independent molecular techniques based on 16S rDNA and rRNA, respectively (Liu and Conrad, 2010, 2011). Aggregates, as close physical contact necessary for DIET, have been found in the culture with ethanol as electron donor after three transfers indicated the possibility of DIET between them. Meantime, strains of *Clostridium* spp. and *Methanosarcina mazei* have been isolated by culture dependent methods. Although the isolation of *Geobacter* species is still on the way, how the aggregates formed and the potential interactions between *Methanosarcina mazei* and *Geobacteraceae* in the aggregates are attracting questions and warrant further investigation.

### Conflict of interest statement

The authors declare that the research was conducted in the absence of any commercial or financial relationships that could be construed as a potential conflict of interest.
