# Supplementary material for: Co-occurrence of Methanosarcina mazei and Geobacteraceae in an iron (III)-reducing enrichment culture
Source: Front Microbiol. 2015 Sep 8;6:941. doi: 10.3389/fmicb.2015.00941 (PMC4562271; doi:10.3389/fmicb.2015.00941)

***Supplementary Material***

**Co-occurrence of *Methanosarcinamazei* and *Geobacteraceae* in an iron(III)-reducing enrichment culture**

Shiling Zheng^1,2†^, Hongxia Zhang^1,2,3†^, Ying Li^1,2,3^, Hua Zhang^1^, Oumei Wang^4^*, Jun Zhang^5^ and Fanghua Liu^1,2^*

^1^ Key Laboratory of Coastal Environmental Processes and Ecological Remediation, Yantai Institute of Coastal Zone Research, Chinese Academy of Sciences , Yantai, China;

^2^ Key Laboratory of Coastal Biology and Biological Resources Utilization, Yantai Institute of Coastal Zone Research, Chinese Academy of Sciences, Yantai, China;

^3^ University of Chinese Academy of Sciences, Beijing, China;

^4^ Key Laboratory for Genetic Hearing Disorders in Shandong, Binzhou Medical University, Yantai, China;

^5^ The College of Life Sciences, Northwest University, Xi’an, China.

^†^Shiling Zheng and Hongxia Zhang have contributed equally to this publication.

*Correspondence:

Fanghua Liu, Yantai Institute of Coastal Zone Research, 17 Chunhui Road, Laishan District, Yantai, Shandong 264003, China. email: fhliu@yic.ac.cn;

or Oumei Wang, Binzhou Medical University, 346 Guanhai Road, Laishan District, Yantai, Shandong 264003, China. email: ziou79@hotmail.com.

Running title: Co-occurrence of *Methanosarcinamazei and Geobacteraceae*

**Key words: Co-occurrence, *Methanosarcinamazei*, *Geobacteraceae*, direct interspecies electron transfer (DIET), iron (III)-reducing microorganisms**

**Supplementary Figures**

**FIGURE S1: OTU diversity of three sediment samples from DNA extracts**. Rarefaction curves for bacteria communities indicating the expected OUT richness of the clone libraries with different sampling efforts. Red, blue and orange represented sediments from site Jh1, Jh2 and Jh3. D, sequences from DNA extract.

**FIGURE S2:Illumina sequencing derived heatmap (A) and relative abundance of bacterial community structures (B) of dominant phyla of three sediment samples(Jh1, Jh2 and Jh3)**.

**FIGURE S1**


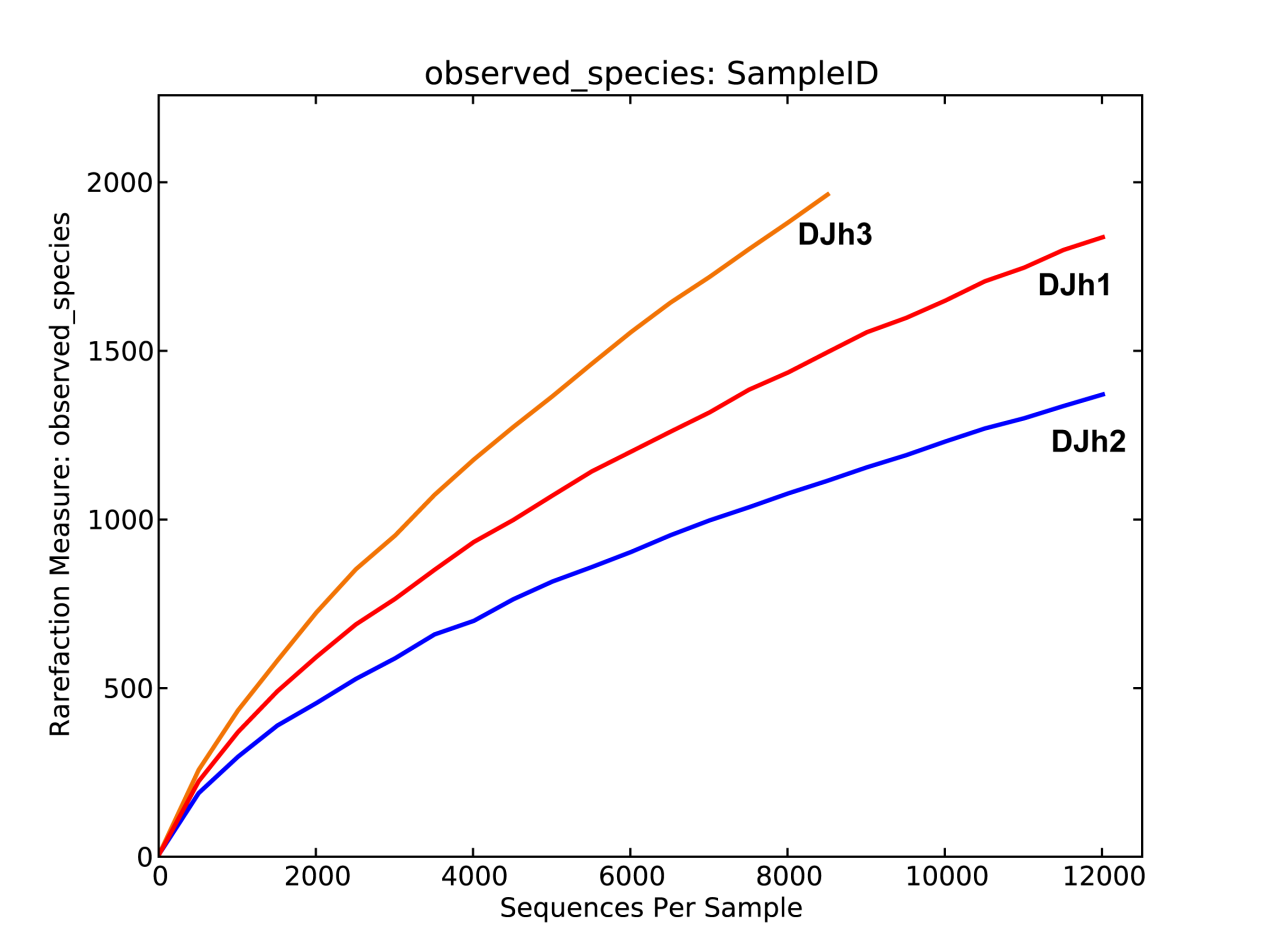


**FIGURE S2**


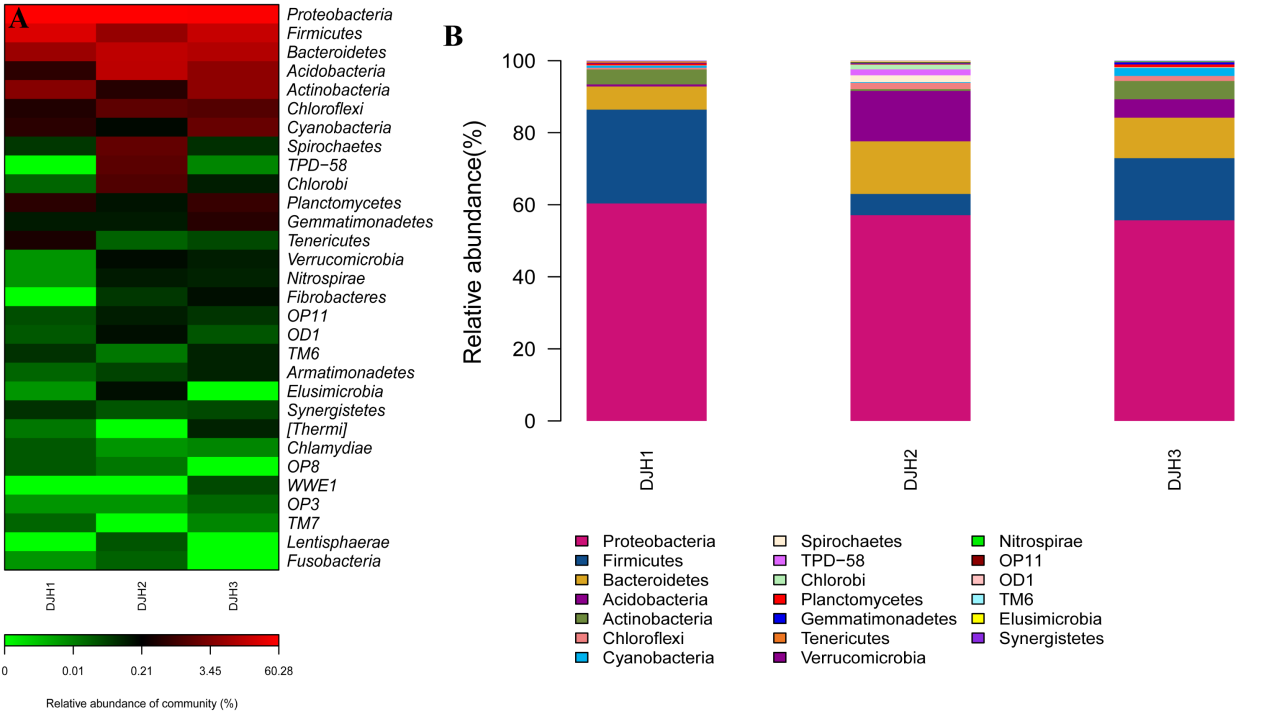

Supplement: Supplementary file 1 [file DataSheet1.DOCX]
